# Anconeus Tongue Approach for Chronic Pediatric Monteggia Lesions: A Case Report

**DOI:** 10.7759/cureus.117807

**Published:** 2026-10-12

**Authors:** Giuseppe Bardellini, Andrea Celli, Luigi Celli

**Affiliations:** 1 Orthopaedics and Traumatology, Hesperia Hospital, Modena, ITA

**Keywords:** anconeus tongue approach, chronic radial head dislocation, monteggia lesion misdiagnosis, radial head reduction, ulnar length restoration

## Abstract

The combination of ulnar fracture and radial head (RH) dislocation gives rise to a set of injuries known as Monteggia lesions. Four weeks after the trauma, pediatric Monteggia lesions are considered chronic and unreduced. In children, the ulnar fracture heals quickly, and the formation of fibrous tissue prevents closed reduction. In such cases, stable RH reduction by means of ulnar osteotomy, correction of ulnar angulation, and ulnar lengthening are usually successful. This case with 20-year follow-up involves a chronic pediatric Monteggia lesion managed by ulnar osteotomy and RH reduction using an anconeus flap approach. This case highlights several key aspects: in delayed presentations, reconstructive surgery must address both the ulnar deformity and the chronic RH dislocation; overcorrection of the ulnar angulation was critical in maintaining the RH reduction; and the anconeus tongue approach affords extensive exposure of both the joints through a single access.

## Introduction

The combination of ulnar fracture and radial head (RH) dislocation gives rise to a set of injuries known as Monteggia lesions. Described by Monteggia in 1814 [1] and by Bado in 1967 [2], these lesions have been divided into four categories depending on the type of RH dislocation. Monteggia lesions are at high risk of being missed (16%-52%) [3-5]. Diagnosis requires careful examination of appropriate X-ray views to visualize the ulnar fracture and the mutual relationships among the radiocapitellar joint (RCJ) surfaces. Computed tomography (CT) with 3D reconstruction provides critical information on the extent of the bony injury, the spatial displacement of the fragments, and RCJ relationships. In the absence of appropriate surgical management, four weeks after the injury, pediatric Monteggia lesions are considered chronic [3-5]. Chronic untreated Monteggia lesions can create several deformities at the humeroradial joint, including cubitus valgus and osteoarthritis. In children, the ulnar fracture heals quickly, and the formation of fibrous tissue prevents closed reduction. In such cases, stable RH reduction can generally be achieved by ulnar osteotomy, correction of ulnar angulation, and ulnar lengthening.

We describe a chronic pediatric Monteggia lesion managed by ulnar osteotomy and RH reduction using the anconeus tongue approach and followed up for 20 years. The anconeus tongue approach theoretically affords safer dissection and greater control of the exposure, thus helping to avoid neurovascular damage to the posterior interosseous nerve. It theoretically also avoids the need for two surgical incisions, providing good access to the RH and the proximal radioulnar joint.

## Case presentation

A five-year-old right-handed girl injured her left elbow 21 years ago. The diagnosis was an undisplaced ulnar fracture. A Monteggia lesion was not suspected, and the RH was not examined (Figure 1).

**Figure 1:**
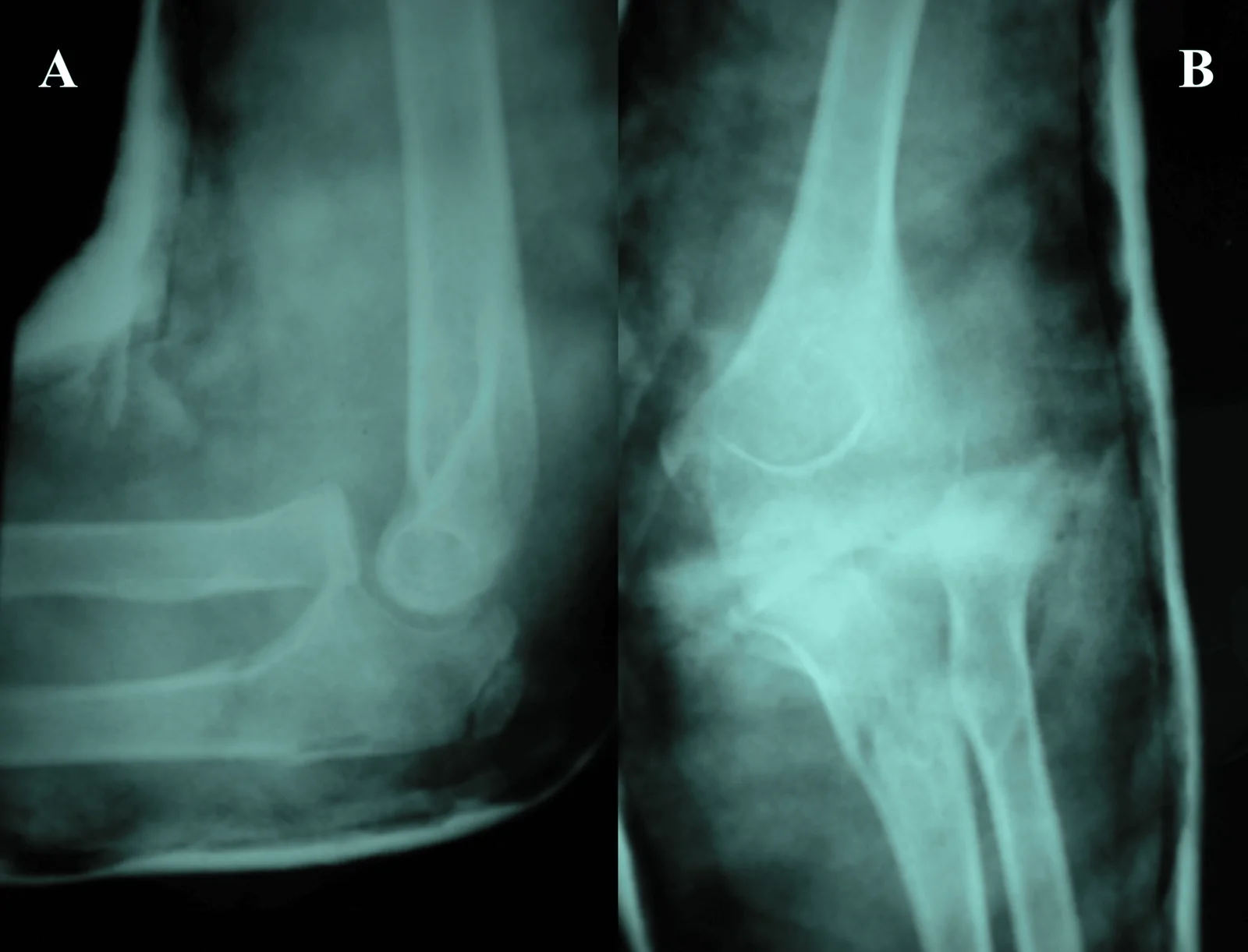
Patient's initial X-rays at the time of trauma: lateral view (A) and antero-posterior view (B). It was diagnosed as an undisplaced ulnar fracture.

Conservative treatment, wearing a cast for four weeks and rehabilitation, failed to restore elbow range of motion. The girl's parents noticed a visible deformity of the forearm. When the patient came to our attention, 12 months after the injury, she had severe functional limitation (flexion-extension arc of 35°, pronation-supination arc of 70°) and extensile deformity of the forearm related to the unreduced RH dislocation and ulna malunion resulting from a missed Monteggia lesion (Figure 2).

**Figure 2:**
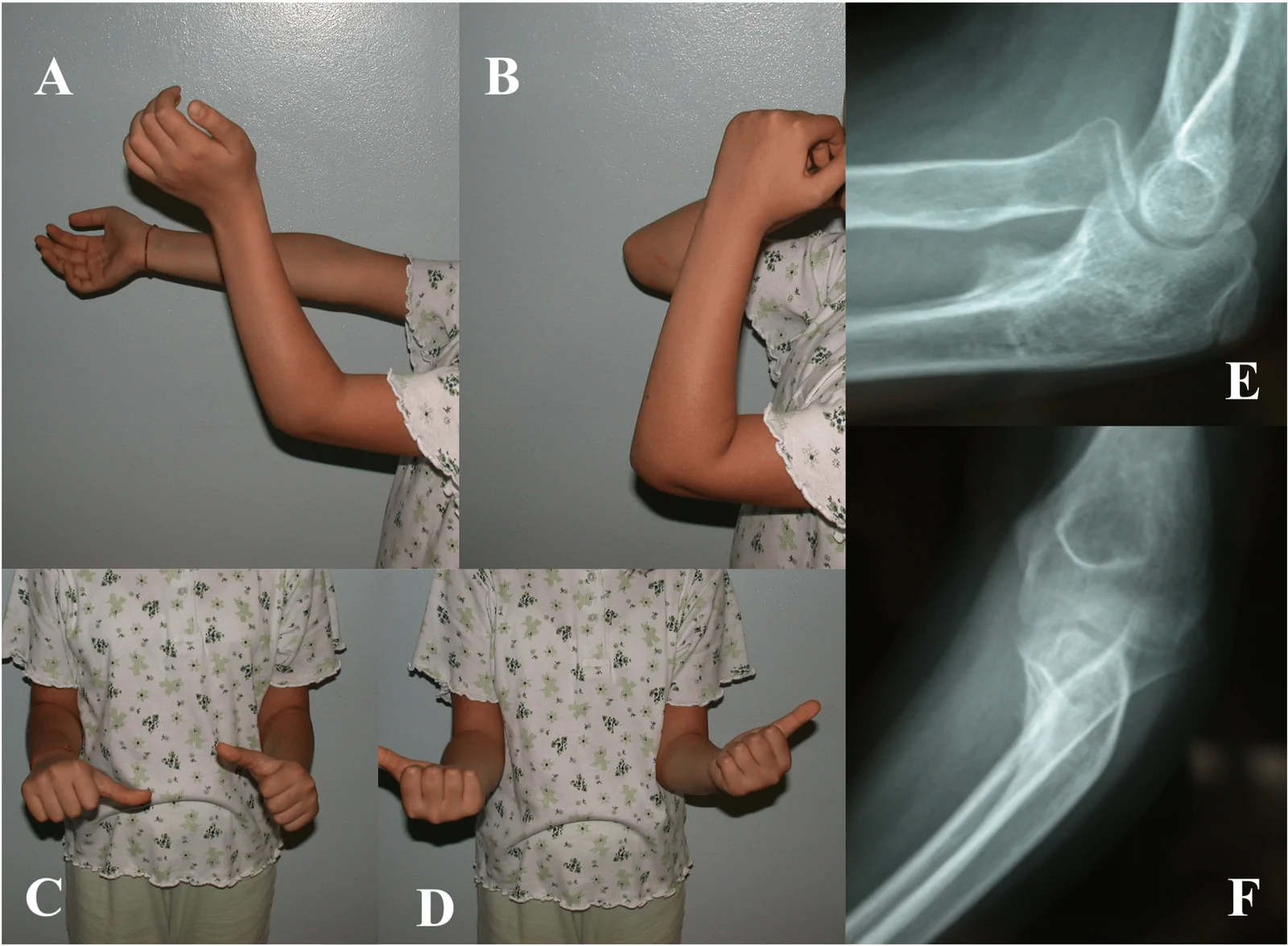
Clinical assessments 12 months after the injury: range of motion in extension (A), in flexion (B), in pronation (C), and in supination (D). Radiographic assessment 12 months after the injury: lateral view (E) and antero-posterior view (F).

Based on the diagnosis and the interval from the trauma, we decided to perform ulnar osteotomy, RH reduction, and re-tensioning of the annular ligament.

Surgical technique

The patient was placed in lateral decubitus, and a tourniquet was used. We performed a posterolateral skin incision proceeding distally from the tip of the olecranon along the posterior border of the ulna to expose the dislocated RH. We raised medial and lateral skin flaps and identified the ulnar nerve proximally, where it emerges under the triceps tendon. We followed the nerve into the cubital tunnel and between the two heads of the flexor carpi ulnaris. Laterally, we dissected the interval between the extensor carpi ulnaris and anconeus muscles and exposed the anconeus. We detached the anconeus tongue from its distal attachment and reflected it off the lateral side of the proximal ulna (Figures 3-4).

**Figure 3:**
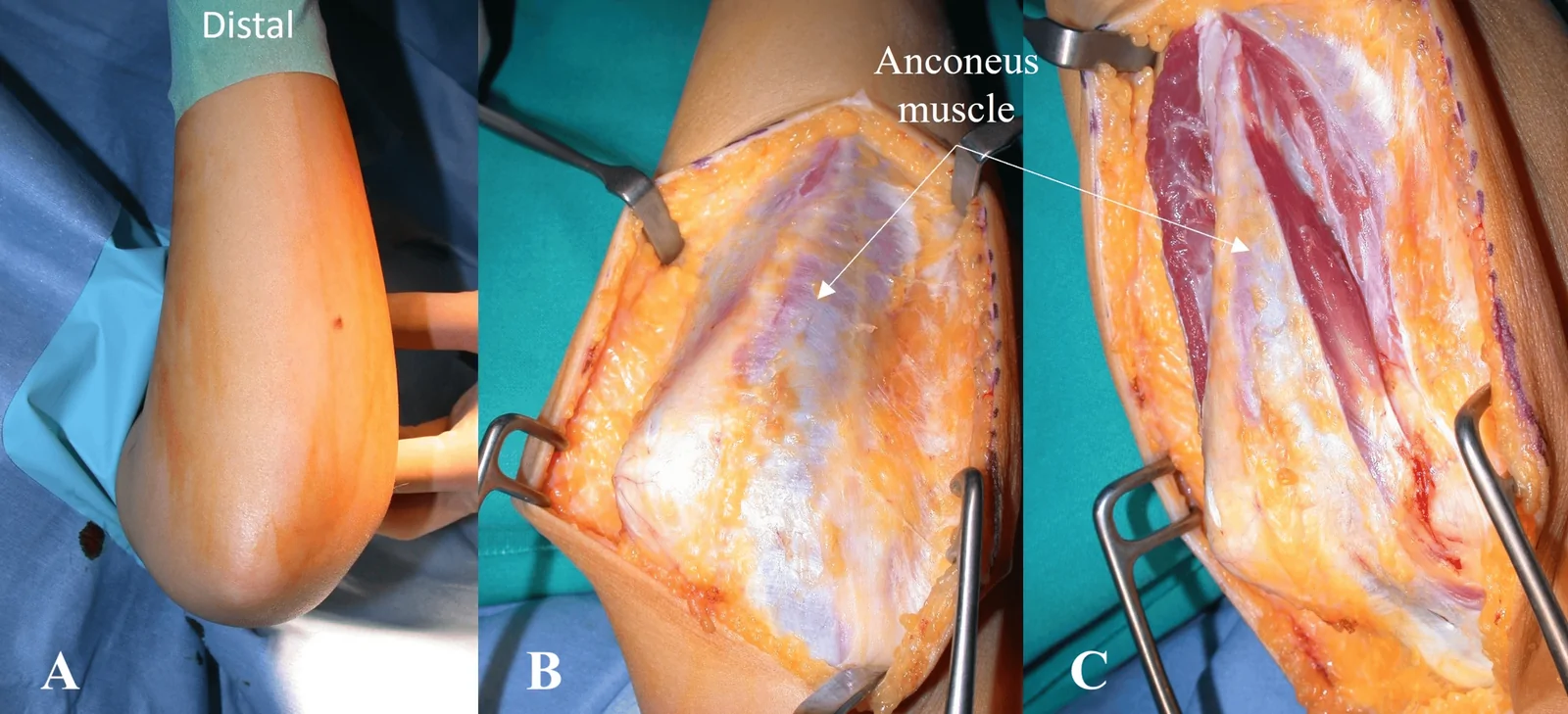
Local deformity of the forearm profile before skin incision (A). Exposure of the interval between the extensor carpi ulnaris and the anconeus muscles (B), and creation of the anconeus tongue (C).

**Figure 4:**
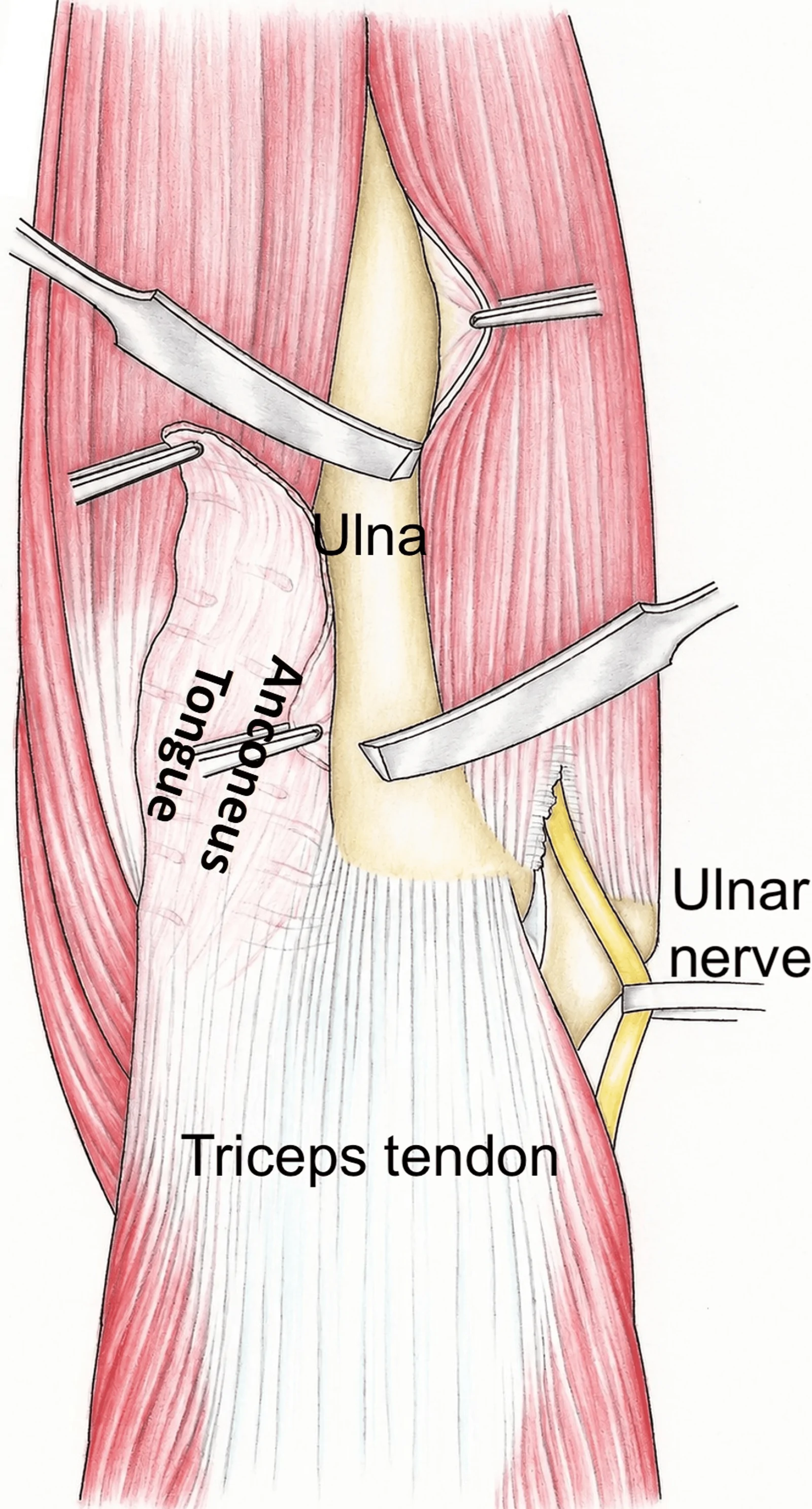
After raising the anconeus flap from its distal attachment, we reflected it off the lateral side of the proximal ulna. The illustration was hand-drawn by a professional medical artist following the authors' instructions.

The dissection was carried out subperiosteally along the radial border of the ulnar crest. We then exposed the lateral collateral ligament (LCL) complex, including the annular ligament. We exposed the proximal radioulnar joint (PRUJ) through the posterior approach used for RH access, opening the annular ligament and the LCL complex (Figure 5).

**Figure 5:**
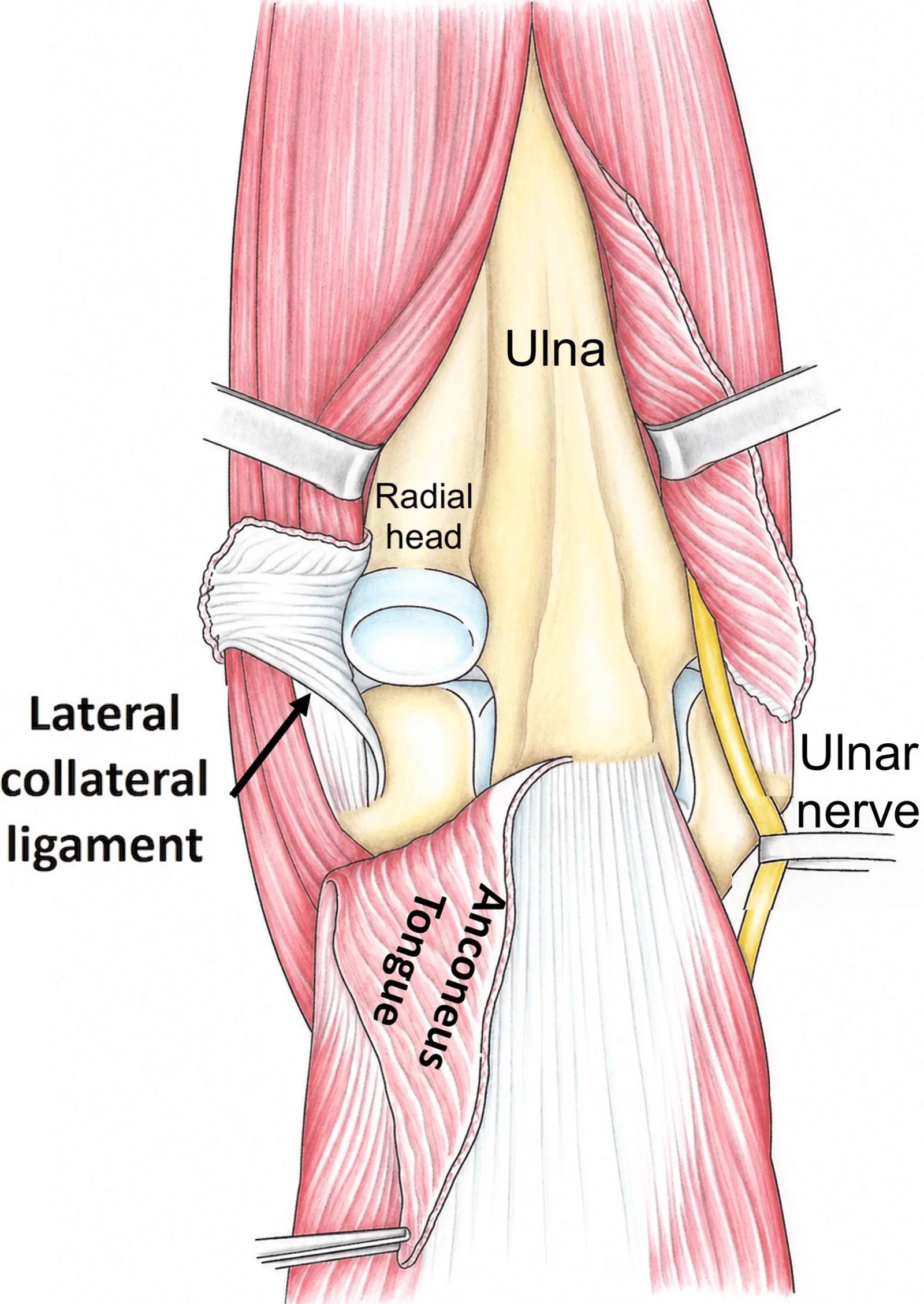
We exposed the proximal radioulnar joint through the posterior approach used for radial head access, opening the annular ligament and the lateral collateral ligament complex. The illustration was hand-drawn by a professional medical artist following the authors' instructions.

We gained access to the RCJ space and removed the scar tissue. Then we prepared the ulna for the osteotomy. Twenty years ago, we used pinning to plan the angulation and level of the cut (Figure 6).

**Figure 6:**
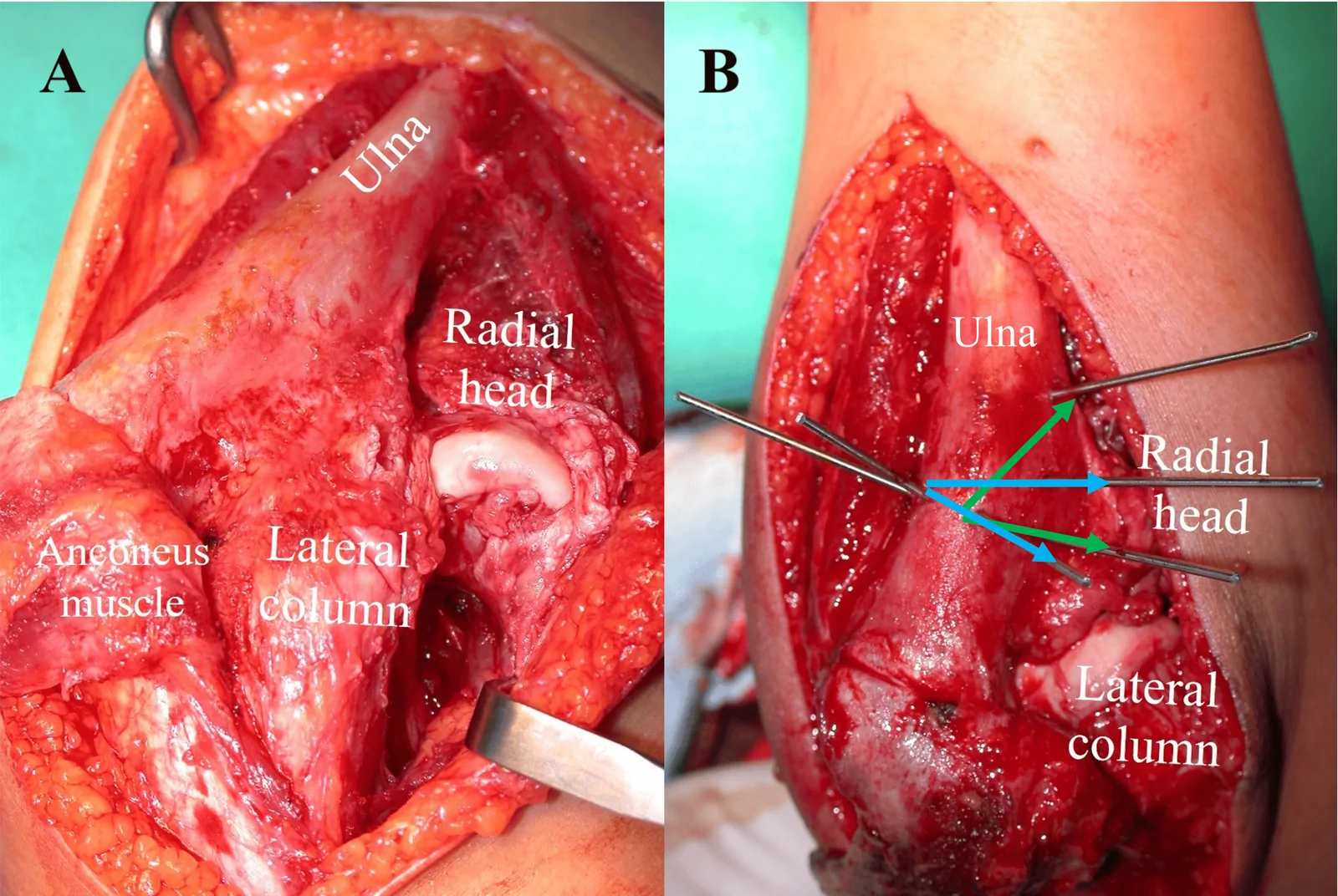
Intra-operative image showing the elevated anconeus tongue, the exposure of the proximal ulna with its deformity, and the radial head dislocation (A). Preparation of the ulnar osteotomy with pinning to plan the angulation and level of the cut (green arrows: sagittal plane osteotomy level, blue arrows: coronal plane osteotomy level) (B).

The ulnar osteotomy is performed extra-articularly, right distal to the coronoid process. The angles and levels of the ulnar osteotomy are planned on pre-operative X-rays and confirmed intra-operatively while assessing the reduction of the radiocapitellar joint. It is an open wedge osteotomy with an inverted V shape and has about 35° of angle on the sagittal plane and about 15° degrees on the coronal plane: this allows obtaining both a dorsal angulation and a varus alignment of the proximal ulna. These two factors are key in restoring the correct radiocapitellar congruity, along with the removal of fibrous tissue interposed between the capitulum humeri and the radial head.

The present approach allows direct visualization of the radial head reduction, while corrective angulation and lengthening of the ulna are progressively performed. This immediate feedback facilitates fine adjustment of osteotomy alignment and contributes to obtaining stable reduction before definitive fixation.

We overcorrected the posterior angulation of the ulna to enhance the stability of the reduction, and we assessed the degree of overcorrection with an intraoperative comparison to the contralateral side. During the osteotomy, we ensured through the same posterior access that the RH did not lose its reduction throughout anconeus reflection and PRUJ exposure.

After careful reduction of the PRUJ, we fixed the ulnar osteotomy with a precontoured dorsal plate (Figure 7).

**Figure 7:**
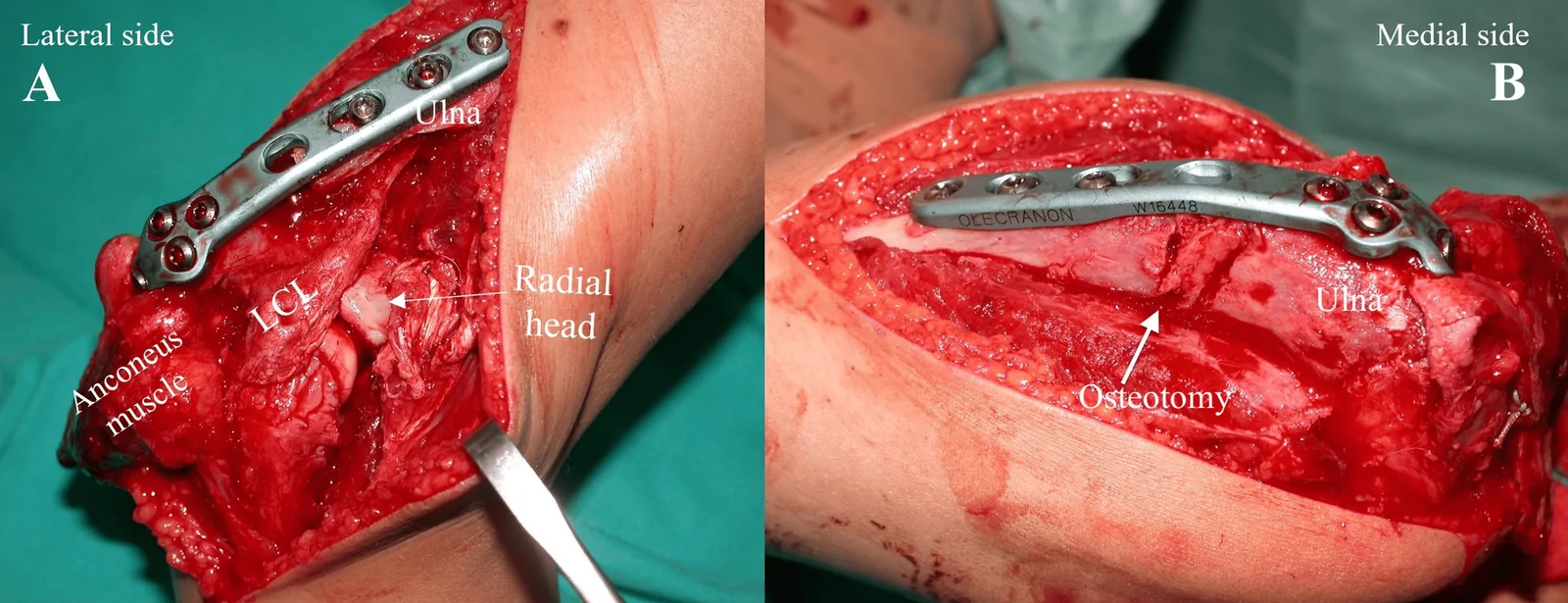
Fixation of the ulnar osteotomy with a precontoured dorsal plate and reconstruction of the annular ligament and the lateral collateral ligament. View from the lateral side (A) and from the medial side (B).

We reconstructed the annular ligament with non-absorbable Krackow locking sutures, tied to the LCL complex while ensuring perfect congruity of the proximal radio-ulnar and radio-capitellar joints. We sutured the anconeus flap back to the ulna and closed the Kocher interval.

There were no intraoperative complications. The patient wore an open cast for four weeks and then began the rehabilitation program. The plate was removed after 10 years, and the patient was followed up for 20 years. Twenty years later, the patient still enjoys a full range of motion in flexion-extension and pronation-supination and has a Mayo Elbow Performance Score [6] of 100 and a Quick Disabilities of the Arm, Shoulder and Hand [7] (QuickDASH) questionnaire score of 2.3. The radiocapitellar joint is congruent, and no particular signs of osteoarthritis are noticeable (Figure 8).

**Figure 8:**
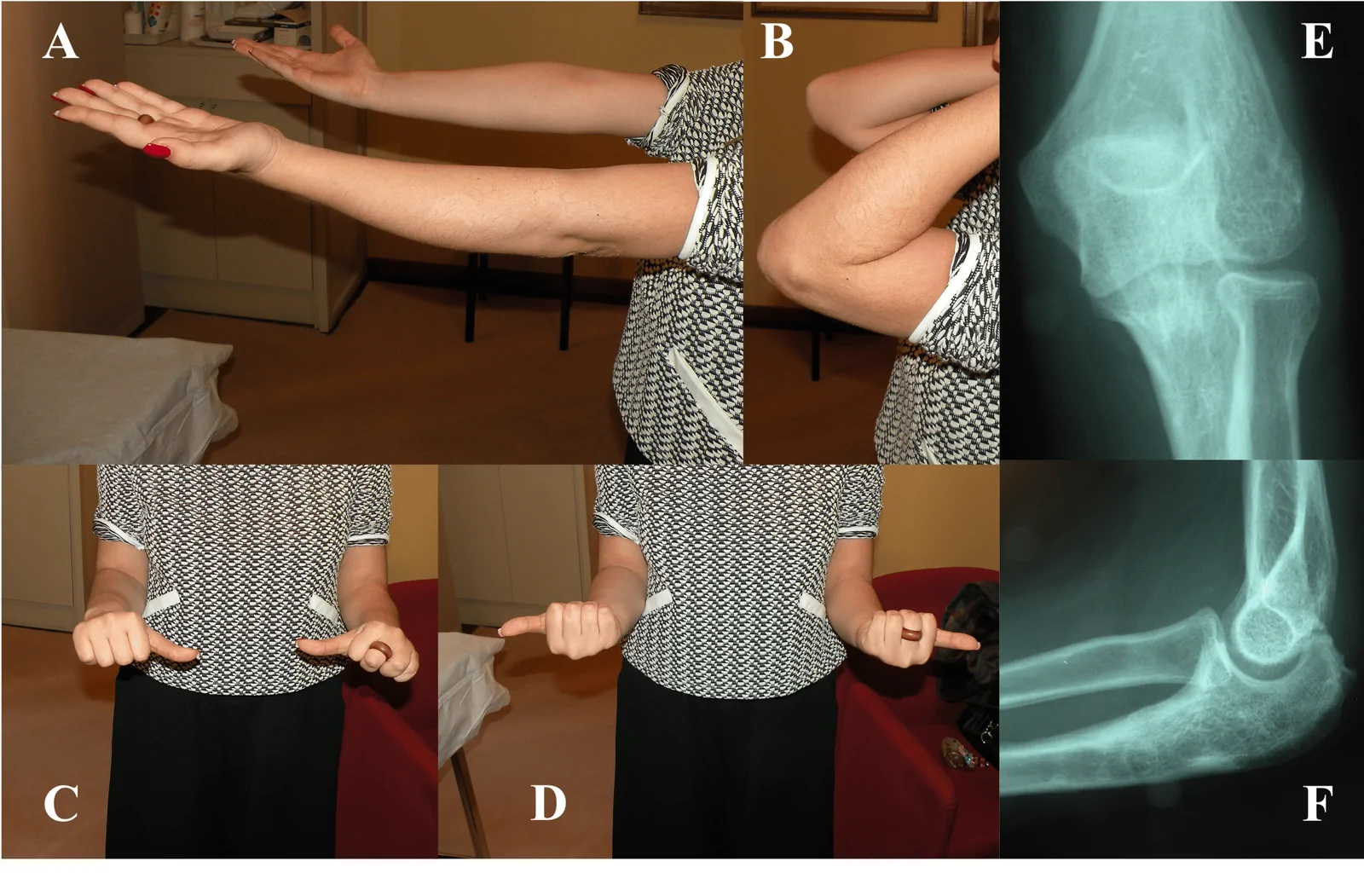
Clinical assessments at 20 years of follow-up: range of motion in extension (A), in flexion (B), in pronation (C), and in supination (D). Radiographic assessment at 20 years: antero-posterior view (E) and lateral view (F).

## Discussion

This case highlights the challenges associated with delayed diagnosis and treatment of a pediatric Monteggia lesion. In our patient, the lack of prompt and appropriate intervention resulted in significant functional impairment and visible deformity. By the time she came to our attention, 12 months after the trauma, she had developed severe range of motion limitations and extensile deformity of the forearm. The surgical approach to these chronic Monteggia lesions involves correcting the ulnar deformity through ulnar osteotomy and reducing the dislocated RH. We combined this technique with re-tensioning of the annular ligament and the LCL complex to restore PRUJ stability. The anconeus tongue approach provided excellent posterior exposure to remove the scar tissue and ensure proper RH reduction throughout the ulnar osteotomy. The dorsal plate, used for osteotomy fixation, provided adequate stability. Meticulous re-tensioning of the annular ligament further contributed to restoring functional anatomy.

The good patient-reported outcomes underscore the long-term effectiveness of our surgical strategy in restoring function and maintaining joint congruency. The radiological assessment confirmed the ulna overcorrection, which contributed to the stability of the RH reduction. The anconeus tongue approach affords extensive exposure of both the PRUJ and the RH through a single access. It provides a direct posterior view of the RH and PRUJ joint and facilitates intraoperative management of the reduction. By obviating the need for two separate incisions, it also theoretically minimizes surgical time and the risk of complications associated with multiple approaches. Anconeus reflection might afford safer dissection and greater control of the exposure, thus helping avoid neurovascular damage, especially to the posterior interosseous nerve, which, following RH dislocation, runs near the RH. Reflection and reattachment of the anconeus muscle preserve its function. This is particularly important in children, where maintaining muscle structure integrity is essential for long-term functional recovery. The surgical exposure adopted in our case differs from the classic Boyd approach, which has traditionally been advocated for complex proximal forearm injuries and chronic Monteggia lesions because it provides extensive exposure of both the proximal radioulnar and radiocapitellar joints through a posterior incision. However, the Boyd approach [3] requires elevation of the anconeus together with the extensor muscle origin and release of the lateral ulnar collateral ligament complex from the ulna, often extending through the supinator. Although this exposure is versatile, it may increase soft-tissue disruption and requires meticulous reconstruction to restore posterolateral elbow stability. Furthermore, the posterior interosseous nerve remains at risk during distal dissection around the supinator, particularly in chronic Monteggia lesions in which normal anatomy is distorted by fibrosis and prolonged RH dislocation. In contrast, the anconeus tongue approach preserves the anconeus as a vascularized muscular flap while providing direct visualization of both the radiocapitellar and proximal radioulnar joints. Reflection of the anconeus from its ulnar insertion creates a broad posterior window that allows scar excision, RH reduction, assessment of reduction throughout ulnar osteotomy, and ligament repair without the need for separate medial or lateral approaches. Preservation of the muscle may also provide a biologically favorable soft-tissue envelope over the reconstructed lateral elbow and may reduce postoperative adhesions. Another potential advantage of this technique is the continuous visualization of the RH during ulnar osteotomy and plate fixation. In conventional lateral approaches, reduction is often checked intermittently, whereas the present exposure enables direct monitoring of RH congruency throughout correction of ulnar length and angulation. This may facilitate fine adjustment of the osteotomy before definitive fixation and reduce the risk of residual subluxation. Nevertheless, the anconeus tongue approach is not without limitations. Compared with the Boyd approach, it is less familiar to most elbow surgeons and requires careful subperiosteal elevation and anatomical reinsertion of the anconeus. In addition, current evidence is limited to isolated reports and small case series, which describe the possibility of accessing the elbow joint with reflection of the anconeus and the lateral part of the triceps tendon [8,9]. No articles reported only the use of the reflection of the anconeus tongue approach, and none of these reports have ever been used for the treatment of a chronic Monteggia fracture-dislocation, precluding definitive conclusions regarding superiority over established posterior or posterolateral approaches. Comparative clinical studies are therefore required to determine whether the theoretical advantages in soft-tissue preservation translate into improved functional outcomes, lower complication rates, or easier revision surgery.

## Conclusions

This case with a 20-year follow-up involves a chronic pediatric Monteggia lesion managed by ulnar osteotomy and RH reduction using an anconeus tongue approach. This case highlights several key aspects: in delayed presentations, reconstructive surgery must address both the ulnar deformity and the chronic RH dislocation; overcorrection of the ulnar angulation can be useful in maintaining the RH reduction; and the anconeus tongue approach affords extensive exposure of both joints through a single access. Further studies are needed to assess the generalizability of our outcomes and the clinical effectiveness of this approach.
